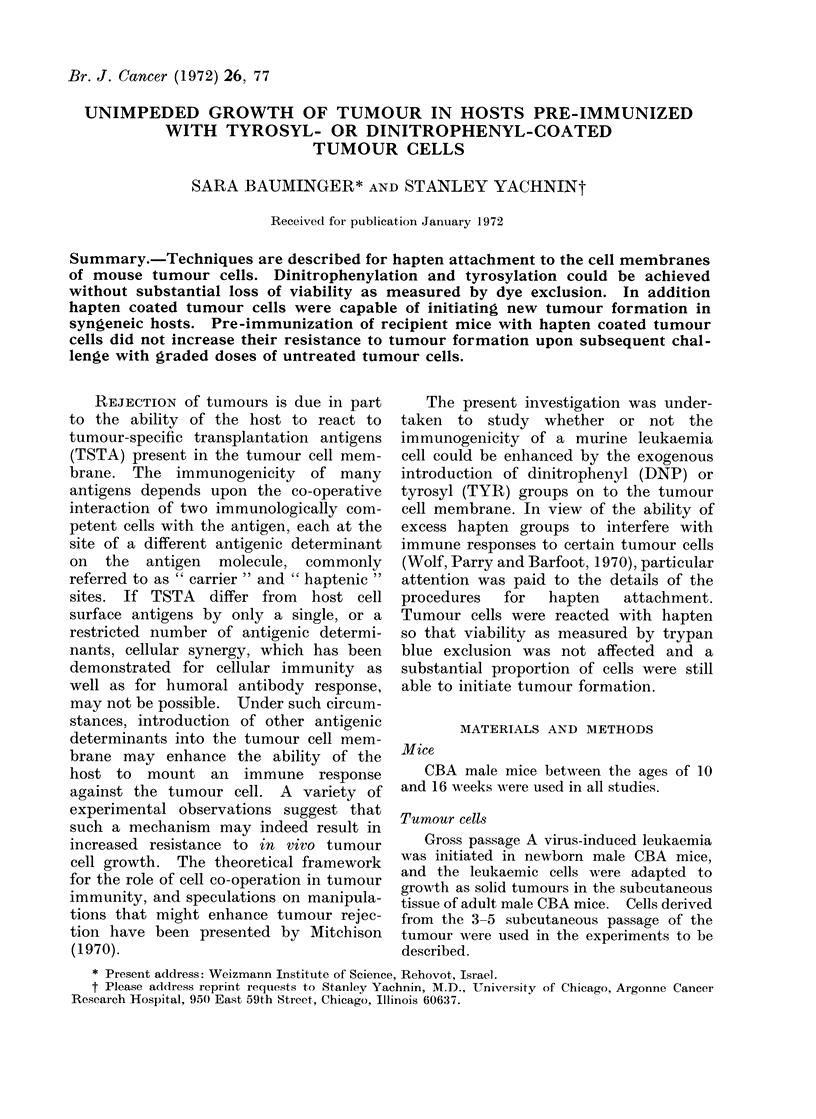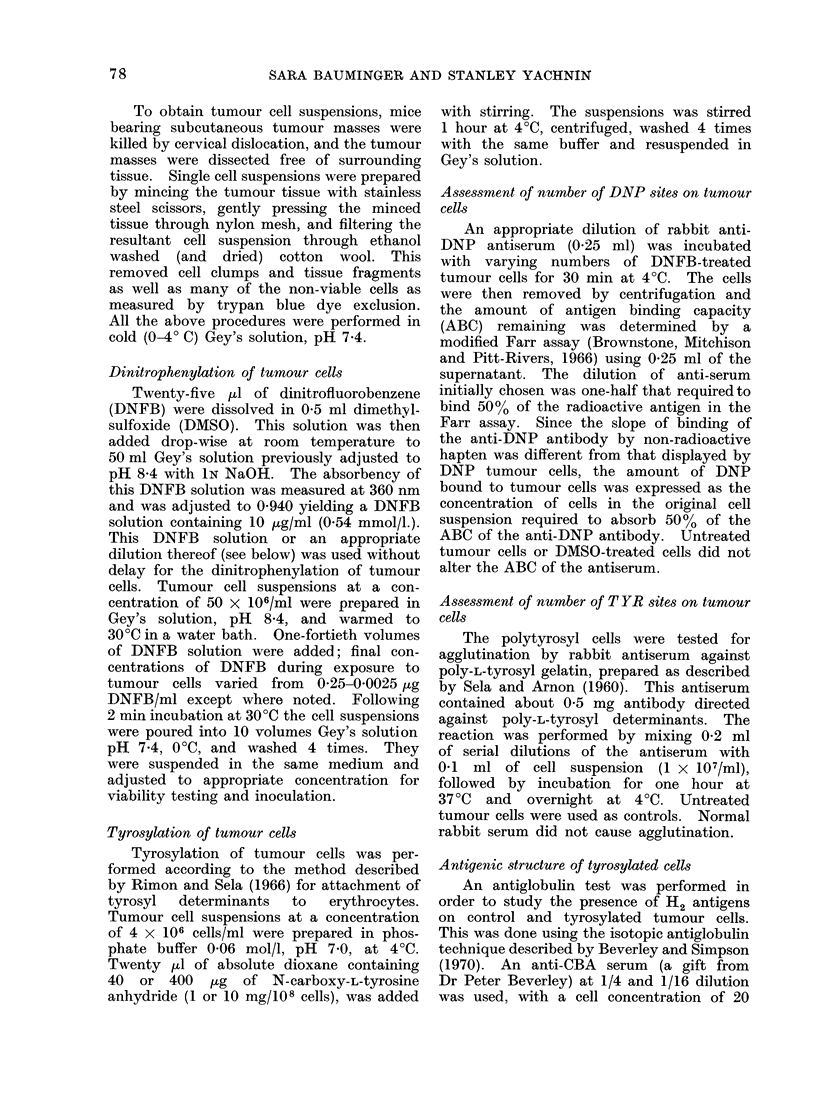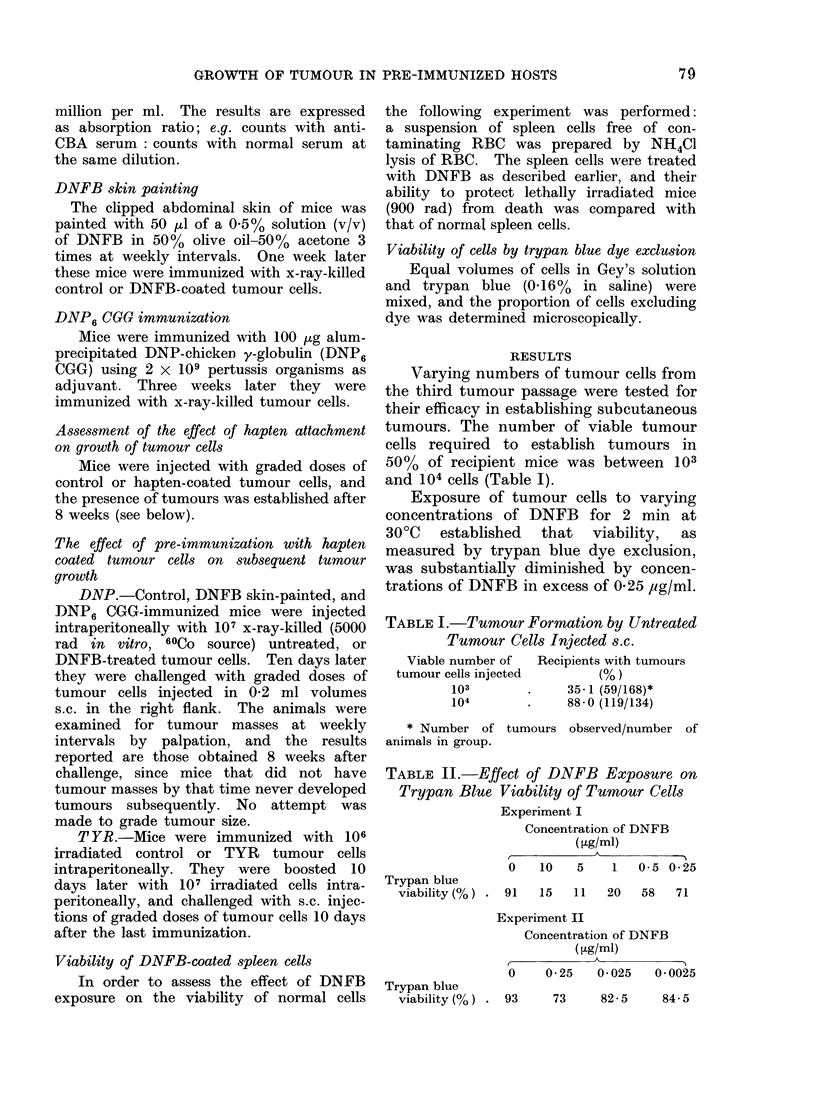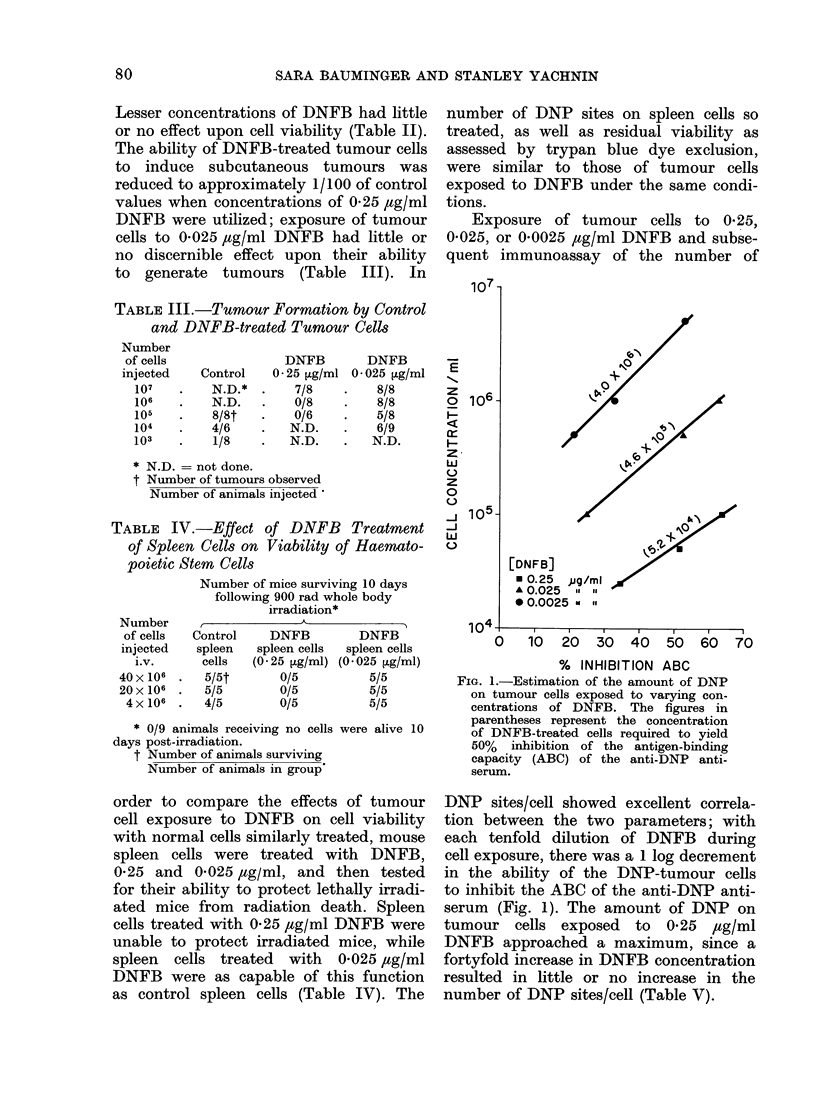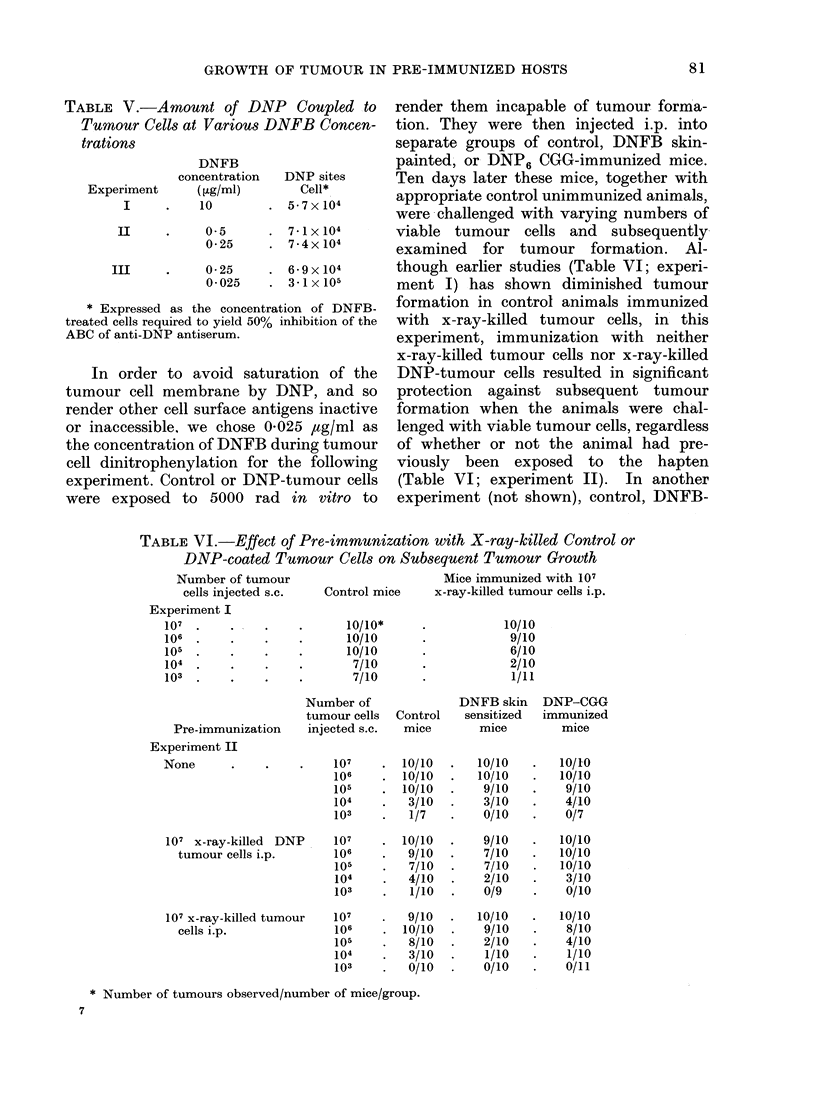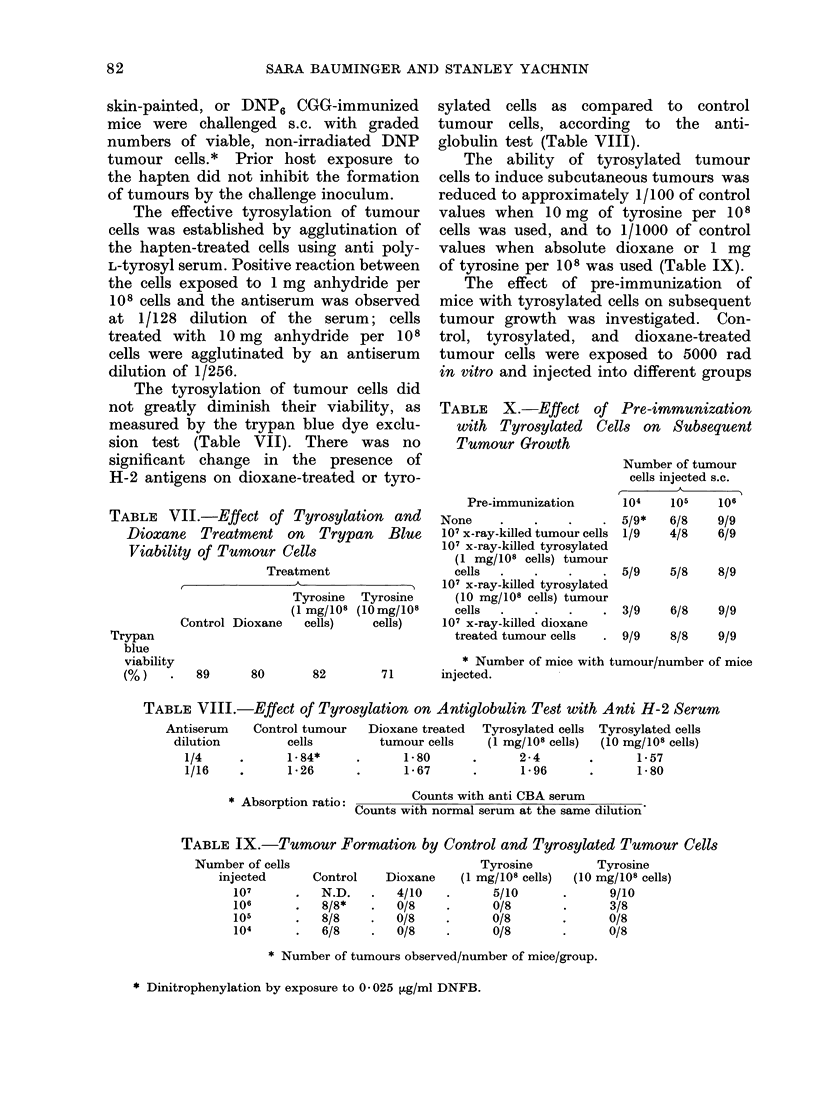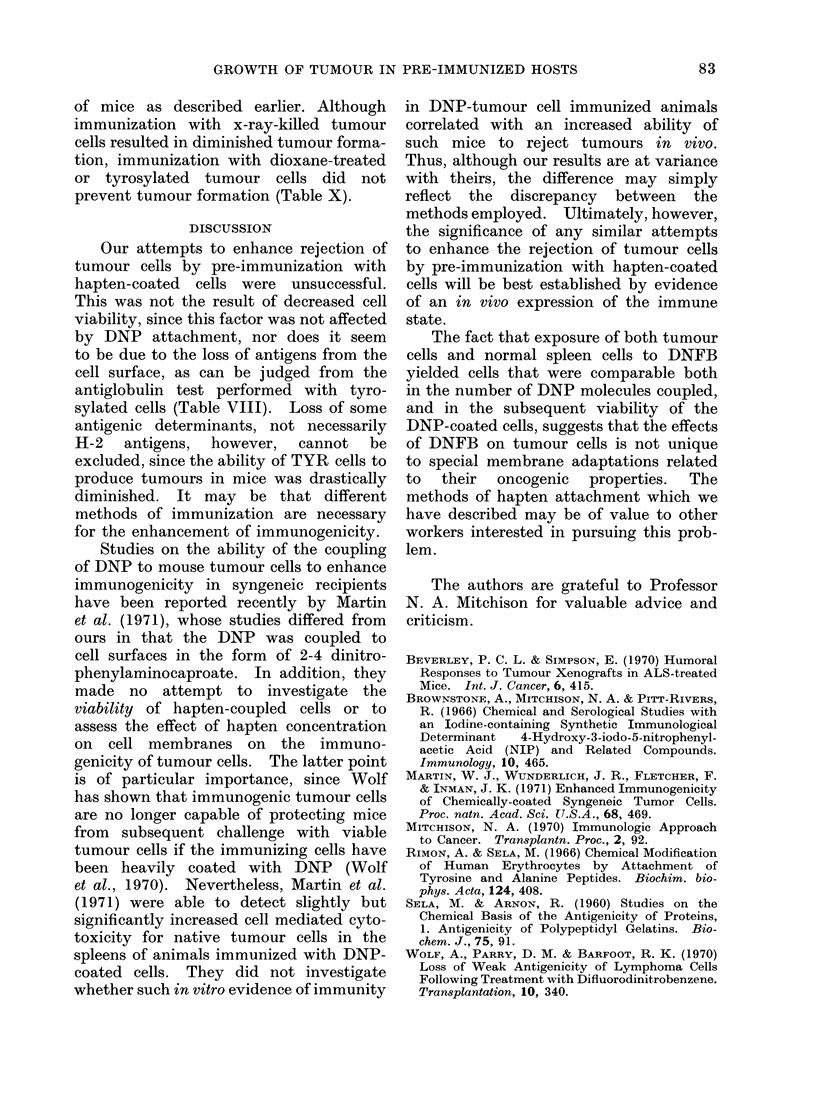# Unimpeded Growth of Tumour in Hosts Pre-immunized with Tyrosyl- or Dinitrophenyl-coated Tumour Cells

**DOI:** 10.1038/bjc.1972.12

**Published:** 1972-04

**Authors:** Sara Bauminger, Stanley Yachnin

## Abstract

Techniques are described for hapten attachment to the cell membranes of mouse tumour cells. Dinitrophenylation and tyrosylation could be achieved without substantial loss of viability as measured by dye exclusion. In addition hapten coated tumour cells were capable of initiating new tumour formation in syngeneic hosts. Pre-immunization of recipient mice with hapten coated tumour cells did not increase their resistance to tumour formation upon subsequent challenge with graded doses of untreated tumour cells.


*Br. J. Cancer* (1972) **26**, 77

### UNIMPEDED GROWTH OF TUMOUR IN HOSTS PRE-IMMUNIZED WITH TYROSYL- OR DINITROPHENYL-COATED TUMOUR CELLS

SARA BAUMINGER* AND STANLEY YACHNIN†

Received for publication January 1972

**Summary.—Techniques are described for hapten attachment to the cell membranes of mouse tumour cells. Dinitrophenylation and tyrosylation could be achieved without substantial loss of viability as measured by dye exclusion. In addition hapten coated tumour cells were capable of initiating new tumour formation in syngeneic hosts. Pre-immunization of recipient mice with hapten coated tumour cells did not increase their resistance to tumour formation upon subsequent challenge with graded doses of untreated tumour cells.**

REJECTION of tumours is due in part to the ability of the host to react to tumour-specific transplantation antigens (TSTA) present in the tumour cell membrane. The immunogenicity of many antigens depends upon the co-operative interaction of two immunologically competent cells with the antigen, each at the site of a different antigenic determinant on the antigen molecule, commonly referred to as " carrier " and " haptenic " sites. If TSTA differ from host cell surface antigens by only a single, or a restricted number of antigenic determinants, cellular synergy, which has been demonstrated for cellular immunity as well as for humoral antibody response, may not be possible. Under such circumstances, introduction of other antigenic determinants into the tumour cell membrane may enhance the ability of the host to mount an immune response against the tumour cell. A variety of experimental observations suggest that such a mechanism may indeed result in increased resistance to *in vivo* tumour cell growth. The theoretical framework for the role of cell co-operation in tumour immunity, and speculations on manipulations that might enhance tumour rejection have been presented by Mitchison (1970).

The present investigation was undertaken to study whether or not the immunogenicity of a murine leukaemia cell could be enhanced by the exogenous introduction of dinitrophenyl (DNP) or tyrosyl (TYR) groups on to the tumour cell membrane. In view of the ability of excess hapten groups to interfere with immune responses to certain tumour cells (Wolf, Parry and Barfoot, 1970), particular attention was paid to the details of the procedures for hapten attachment. Tumour cells were reacted with hapten so that viability as measured by trypan blue exclusion was not affected and a substantial proportion of cells were still able to initiate tumour formation.

### MATERIALS AND METHODS

### *Mice*

CBA male mice between the ages of 10 and 16 weeks were used in all studies.

### *Tumour cells*

Gross passage A virus-induced leukaemia was initiated in newborn male CBA mice, and the leukaemic cells were adapted to growth as solid tumours in the subcutaneous tissue of adult male CBA mice. Cells derived from the 3–5 subcutaneous passage of the tumour were used in the experiments to be described.

* Present address: Weizmann Institute of Science, Rehovot, Israel.
† Please address reprint requests to Stanley Yachnin, M.D., University of Chicago, Argonne Cancer Research Hospital, 950 East 59th Street, Chicago, Illinois 60637.

To obtain tumour cell suspensions, mice bearing subcutaneous tumour masses were killed by cervical dislocation, and the tumour masses were dissected free of surrounding tissue. Single cell suspensions were prepared by mincing the tumour tissue with stainless steel scissors, gently pressing the minced tissue through nylon mesh, and filtering the resultant cell suspension through ethanol washed (and dried) cotton wool. This removed cell clumps and tissue fragments as well as many of the non-viable cells as measured by trypan blue dye exclusion. All the above procedures were performed in cold (0–4° C) Gey's solution, pH 7·4.

*Dinitrophenylation of tumour cells*

Twenty-five $\mu$l of dinitrofluorobenzene (DNFB) were dissolved in 0·5 ml dimethylsulfoxide (DMSO). This solution was then added drop-wise at room temperature to 50 ml Gey's solution previously adjusted to pH 8·4 with 1N NaOH. The absorbency of this DNFB solution was measured at 360 nm and was adjusted to 0·940 yielding a DNFB solution containing 10 $\mu$g/ml (0·54 mmol/l.). This DNFB solution or an appropriate dilution thereof (see below) was used without delay for the dinitrophenylation of tumour cells. Tumour cell suspensions at a concentration of $50 \times 10^6$/ml were prepared in Gey's solution, pH 8·4, and warmed to 30°C in a water bath. One-fortieth volumes of DNFB solution were added; final concentrations of DNFB during exposure to tumour cells varied from 0·25–0·0025 $\mu$g DNFB/ml except where noted. Following 2 min incubation at 30°C the cell suspensions were poured into 10 volumes Gey's solution pH 7·4, 0°C, and washed 4 times. They were suspended in the same medium and adjusted to appropriate concentration for viability testing and inoculation.

*Tyrosylation of tumour cells*

Tyrosylation of tumour cells was performed according to the method described by Rimon and Sela (1966) for attachment of tyrosyl determinants to erythrocytes. Tumour cell suspensions at a concentration of $4 \times 10^6$ cells/ml were prepared in phosphate buffer 0·06 mol/l, pH 7·0, at 4°C. Twenty $\mu$l of absolute dioxane containing 40 or 400 $\mu$g of N-carboxy-L-tyrosine anhydride (1 or 10 mg/$10^8$ cells), was added with stirring. The suspensions was stirred 1 hour at 4°C, centrifuged, washed 4 times with the same buffer and resuspended in Gey's solution.

*Assessment of number of DNP sites on tumour cells*

An appropriate dilution of rabbit anti-DNP antiserum (0·25 ml) was incubated with varying numbers of DNFB-treated tumour cells for 30 min at 4°C. The cells were then removed by centrifugation and the amount of antigen binding capacity (ABC) remaining was determined by a modified Farr assay (Brownstone, Mitchison and Pitt-Rivers, 1966) using 0·25 ml of the supernatant. The dilution of anti-serum initially chosen was one-half that required to bind 50% of the radioactive antigen in the Farr assay. Since the slope of binding of the anti-DNP antibody by non-radioactive hapten was different from that displayed by DNP tumour cells, the amount of DNP bound to tumour cells was expressed as the concentration of cells in the original cell suspension required to absorb 50% of the ABC of the anti-DNP antibody. Untreated tumour cells or DMSO-treated cells did not alter the ABC of the antiserum.

*Assessment of number of TYR sites on tumour cells*

The polytyrosyl cells were tested for agglutination by rabbit antiserum against poly-L-tyrosyl gelatin, prepared as described by Sela and Arnon (1960). This antiserum contained about 0·5 mg antibody directed against poly-L-tyrosyl determinants. The reaction was performed by mixing 0·2 ml of serial dilutions of the antiserum with 0·1 ml of cell suspension ($1 \times 10^7$/ml), followed by incubation for one hour at 37°C and overnight at 4°C. Untreated tumour cells were used as controls. Normal rabbit serum did not cause agglutination.

*Antigenic structure of tyrosylated cells*

An antiglobulin test was performed in order to study the presence of $H_2$ antigens on control and tyrosylated tumour cells. This was done using the isotopic antiglobulin technique described by Beverley and Simpson (1970). An anti-CBA serum (a gift from Dr Peter Beverley) at 1/4 and 1/16 dilution was used, with a cell concentration of 20

million per ml. The results are expressed as absorption ratio; *e.g.* counts with anti-CBA serum : counts with normal serum at the same dilution.

*DNFB skin painting*

The clipped abdominal skin of mice was painted with 50 $\mu$l of a 0·5% solution (v/v) of DNFB in 50% olive oil–50% acetone 3 times at weekly intervals. One week later these mice were immunized with x-ray-killed control or DNFB-coated tumour cells.

*$DNP_6$ CGG immunization*

Mice were immunized with 100 $\mu$g alum-precipitated DNP-chicken $\gamma$-globulin ($DNP_6$ CGG) using $2 \times 10^9$ pertussis organisms as adjuvant. Three weeks later they were immunized with x-ray-killed tumour cells.

*Assessment of the effect of hapten attachment on growth of tumour cells*

Mice were injected with graded doses of control or hapten-coated tumour cells, and the presence of tumours was established after 8 weeks (see below).

*The effect of pre-immunization with hapten coated tumour cells on subsequent tumour growth*

*DNP.*—Control, DNFB skin-painted, and $DNP_6$ CGG-immunized mice were injected intraperitoneally with $10^7$ x-ray-killed (5000 rad *in vitro*, $^{60}$Co source) untreated, or DNFB-treated tumour cells. Ten days later they were challenged with graded doses of tumour cells injected in 0·2 ml volumes s.c. in the right flank. The animals were examined for tumour masses at weekly intervals by palpation, and the results reported are those obtained 8 weeks after challenge, since mice that did not have tumour masses by that time never developed tumours subsequently. No attempt was made to grade tumour size.

*TYR.*—Mice were immunized with $10^6$ irradiated control or TYR tumour cells intraperitoneally. They were boosted 10 days later with $10^7$ irradiated cells intraperitoneally, and challenged with s.c. injections of graded doses of tumour cells 10 days after the last immunization.

*Viability of DNFB-coated spleen cells*

In order to assess the effect of DNFB exposure on the viability of normal cells the following experiment was performed: a suspension of spleen cells free of contaminating RBC was prepared by $NH_4Cl$ lysis of RBC. The spleen cells were treated with DNFB as described earlier, and their ability to protect lethally irradiated mice (900 rad) from death was compared with that of normal spleen cells.

*Viability of cells by trypan blue dye exclusion*

Equal volumes of cells in Gey's solution and trypan blue (0·16% in saline) were mixed, and the proportion of cells excluding dye was determined microscopically.

### RESULTS

Varying numbers of tumour cells from the third tumour passage were tested for their efficacy in establishing subcutaneous tumours. The number of viable tumour cells required to establish tumours in 50% of recipient mice was between $10^3$ and $10^4$ cells (Table I).

Exposure of tumour cells to varying concentrations of DNFB for 2 min at 30°C established that viability, as measured by trypan blue dye exclusion, was substantially diminished by concentrations of DNFB in excess of 0·25 $\mu$g/ml.

TABLE I.—*Tumour Formation by Untreated Tumour Cells Injected s.c.*

| Viable number of tumour cells injected | Recipients with tumours (%) |
|---|---|
| $10^3$ | 35·1 (59/168)* |
| $10^4$ | 88·0 (119/134) |

* Number of tumours observed/number of animals in group.

TABLE II.—*Effect of DNFB Exposure on Trypan Blue Viability of Tumour Cells*

Experiment I

| | Concentration of DNFB (µg/ml) | | | | | |
|---|---|---|---|---|---|---|
| | 0 | 10 | 5 | 1 | 0·5 | 0·25 |
| Trypan blue viability (%) | 91 | 15 | 11 | 20 | 58 | 71 |

Experiment II

| | Concentration of DNFB (µg/ml) | | | |
|---|---|---|---|---|
| | 0 | 0·25 | 0·025 | 0·0025 |
| Trypan blue viability (%) | 93 | 73 | 82·5 | 84·5 |

Lesser concentrations of DNFB had little or no effect upon cell viability (Table II). The ability of DNFB-treated tumour cells to induce subcutaneous tumours was reduced to approximately 1/100 of control values when concentrations of 0·25 μg/ml DNFB were utilized; exposure of tumour cells to 0·025 μg/ml DNFB had little or no discernible effect upon their ability to generate tumours (Table III). In order to compare the effects of tumour cell exposure to DNFB on cell viability with normal cells similarly treated, mouse spleen cells were treated with DNFB, 0·25 and 0·025 μg/ml, and then tested for their ability to protect lethally irradiated mice from radiation death. Spleen cells treated with 0·25 μg/ml DNFB were unable to protect irradiated mice, while spleen cells treated with 0·025 μg/ml DNFB were as capable of this function as control spleen cells (Table IV). The number of DNP sites on spleen cells so treated, as well as residual viability as assessed by trypan blue dye exclusion, were similar to those of tumour cells exposed to DNFB under the same conditions.

TABLE III.—*Tumour Formation by Control and DNFB-treated Tumour Cells*

| Number of cells injected | Control | DNFB 0·25 μg/ml | DNFB 0·025 μg/ml |
|---|---|---|---|
| $10^7$ | N.D.* | 7/8 | 8/8 |
| $10^6$ | N.D. | 0/8 | 8/8 |
| $10^5$ | 8/8† | 0/6 | 5/8 |
| $10^4$ | 4/6 | N.D. | 6/9 |
| $10^3$ | 1/8 | N.D. | N.D. |

* N.D. = not done.
† Number of tumours observed / Number of animals injected.

TABLE IV.—*Effect of DNFB Treatment of Spleen Cells on Viability of Haematopoietic Stem Cells*

| Number of cells injected i.v. | Number of mice surviving 10 days following 900 rad whole body irradiation* | | |
|---|---|---|---|
| | Control spleen cells | DNFB spleen cells (0·25 μg/ml) | DNFB spleen cells (0·025 μg/ml) |
| $40\times10^6$ | 5/5† | 0/5 | 5/5 |
| $20\times10^6$ | 5/5 | 0/5 | 5/5 |
| $4\times10^6$ | 4/5 | 0/5 | 5/5 |

* 0/9 animals receiving no cells were alive 10 days post-irradiation.
† Number of animals surviving / Number of animals in group.

Exposure of tumour cells to 0·25, 0·025, or 0·0025 μg/ml DNFB and subsequent immunoassay of the number of DNP sites/cell showed excellent correlation between the two parameters; with each tenfold dilution of DNFB during cell exposure, there was a 1 log decrement in the ability of the DNP-tumour cells to inhibit the ABC of the anti-DNP antiserum (Fig. 1). The amount of DNP on tumour cells exposed to 0·25 μg/ml DNFB approached a maximum, since a fortyfold increase in DNFB concentration resulted in little or no increase in the number of DNP sites/cell (Table V).

FIG. 1.—Estimation of the amount of DNP on tumour cells exposed to varying concentrations of DNFB. The figures in parentheses represent the concentration of DNFB-treated cells required to yield 50% inhibition of the antigen-binding capacity (ABC) of the anti-DNP antiserum.

TABLE V.—*Amount of DNP Coupled to Tumour Cells at Various DNFB Concentrations*

| Experiment | DNFB concentration (μg/ml) | DNP sites Cell* |
|---|---|---|
| I | 10 | $5 \cdot 7 \times 10^4$ |
| II | 0·5 | $7 \cdot 1 \times 10^4$ |
| | 0·25 | $7 \cdot 4 \times 10^4$ |
| III | 0·25 | $6 \cdot 9 \times 10^4$ |
| | 0·025 | $3 \cdot 1 \times 10^5$ |

* Expressed as the concentration of DNFB-treated cells required to yield 50% inhibition of the ABC of anti-DNP antiserum.

In order to avoid saturation of the tumour cell membrane by DNP, and so render other cell surface antigens inactive or inaccessible, we chose 0·025 μg/ml as the concentration of DNFB during tumour cell dinitrophenylation for the following experiment. Control or DNP-tumour cells were exposed to 5000 rad *in vitro* to render them incapable of tumour formation. They were then injected i.p. into separate groups of control, DNFB skin-painted, or $DNP_6$ CGG-immunized mice. Ten days later these mice, together with appropriate control unimmunized animals, were challenged with varying numbers of viable tumour cells and subsequently examined for tumour formation. Although earlier studies (Table VI; experiment I) has shown diminished tumour formation in control animals immunized with x-ray-killed tumour cells, in this experiment, immunization with neither x-ray-killed tumour cells nor x-ray-killed DNP-tumour cells resulted in significant protection against subsequent tumour formation when the animals were challenged with viable tumour cells, regardless of whether or not the animal had previously been exposed to the hapten (Table VI; experiment II). In another experiment (not shown), control, DNFB-

TABLE VI.—*Effect of Pre-immunization with X-ray-killed Control or DNP-coated Tumour Cells on Subsequent Tumour Growth*

| Number of tumour cells injected s.c. | Control mice | Mice immunized with $10^7$ x-ray-killed tumour cells i.p. |
|---|---|---|
| Experiment I | | |
| $10^7$ | 10/10* | 10/10 |
| $10^6$ | 10/10 | 9/10 |
| $10^5$ | 10/10 | 6/10 |
| $10^4$ | 7/10 | 2/10 |
| $10^3$ | 7/10 | 1/11 |

| Pre-immunization | Number of tumour cells injected s.c. | Control mice | DNFB skin sensitized mice | DNP–CGG immunized mice |
|---|---|---|---|---|
| Experiment II | | | | |
| None | $10^7$ | 10/10 | 10/10 | 10/10 |
| | $10^6$ | 10/10 | 10/10 | 10/10 |
| | $10^5$ | 10/10 | 9/10 | 9/10 |
| | $10^4$ | 3/10 | 3/10 | 4/10 |
| | $10^3$ | 1/7 | 0/10 | 0/7 |
| $10^7$ x-ray-killed DNP tumour cells i.p. | $10^7$ | 10/10 | 9/10 | 10/10 |
| | $10^6$ | 9/10 | 7/10 | 10/10 |
| | $10^5$ | 7/10 | 7/10 | 10/10 |
| | $10^4$ | 4/10 | 2/10 | 3/10 |
| | $10^3$ | 1/10 | 0/9 | 0/10 |
| $10^7$ x-ray-killed tumour cells i.p. | $10^7$ | 9/10 | 10/10 | 10/10 |
| | $10^6$ | 10/10 | 9/10 | 8/10 |
| | $10^5$ | 8/10 | 2/10 | 4/10 |
| | $10^4$ | 3/10 | 1/10 | 1/10 |
| | $10^3$ | 0/10 | 0/10 | 0/11 |

* Number of tumours observed/number of mice/group.

skin-painted, or $DNP_6$ CGG-immunized mice were challenged s.c. with graded numbers of viable, non-irradiated DNP tumour cells.* Prior host exposure to the hapten did not inhibit the formation of tumours by the challenge inoculum.

The effective tyrosylation of tumour cells was established by agglutination of the hapten-treated cells using anti poly-L-tyrosyl serum. Positive reaction between the cells exposed to 1 mg anhydride per $10^8$ cells and the antiserum was observed at 1/128 dilution of the serum; cells treated with 10 mg anhydride per $10^8$ cells were agglutinated by an antiserum dilution of 1/256.

The tyrosylation of tumour cells did not greatly diminish their viability, as measured by the trypan blue dye exclusion test (Table VII). There was no significant change in the presence of H-2 antigens on dioxane-treated or tyrosylated cells as compared to control tumour cells, according to the antiglobulin test (Table VIII).

The ability of tyrosylated tumour cells to induce subcutaneous tumours was reduced to approximately 1/100 of control values when 10 mg of tyrosine per $10^8$ cells was used, and to 1/1000 of control values when absolute dioxane or 1 mg of tyrosine per $10^8$ was used (Table IX).

The effect of pre-immunization of mice with tyrosylated cells on subsequent tumour growth was investigated. Control, tyrosylated, and dioxane-treated tumour cells were exposed to 5000 rad *in vitro* and injected into different groups

TABLE VII.—*Effect of Tyrosylation and Dioxane Treatment on Trypan Blue Viability of Tumour Cells*

| | Treatment | | | |
|---|---|---|---|---|
| | Control | Dioxane | Tyrosine (1 mg/$10^8$ cells) | Tyrosine (10 mg/$10^8$ cells) |
| Trypan blue viability (%) | 89 | 80 | 82 | 71 |

TABLE X.—*Effect of Pre-immunization with Tyrosylated Cells on Subsequent Tumour Growth*

| Pre-immunization | Number of tumour cells injected s.c. | | |
|---|---|---|---|
| | $10^4$ | $10^5$ | $10^6$ |
| None | 5/9* | 6/8 | 9/9 |
| $10^7$ x-ray-killed tumour cells | 1/9 | 4/8 | 6/9 |
| $10^7$ x-ray-killed tyrosylated (1 mg/$10^8$ cells) tumour cells | 5/9 | 5/8 | 8/9 |
| $10^7$ x-ray-killed tyrosylated (10 mg/$10^8$ cells) tumour cells | 3/9 | 6/8 | 9/9 |
| $10^7$ x-ray-killed dioxane treated tumour cells | 9/9 | 8/8 | 9/9 |

* Number of mice with tumour/number of mice injected.

TABLE VIII.—*Effect of Tyrosylation on Antiglobulin Test with Anti H-2 Serum*

| Antiserum dilution | Control tumour cells | Dioxane treated tumour cells | Tyrosylated cells (1 mg/$10^8$ cells) | Tyrosylated cells (10 mg/$10^8$ cells) |
|---|---|---|---|---|
| 1/4 | 1·84* | 1·80 | 2·4 | 1·57 |
| 1/16 | 1·26 | 1·67 | 1·96 | 1·80 |

$$* \text{ Absorption ratio: } \frac{\text{Counts with anti CBA serum}}{\text{Counts with normal serum at the same dilution}}.$$

TABLE IX.—*Tumour Formation by Control and Tyrosylated Tumour Cells*

| Number of cells injected | Control | Dioxane | Tyrosine (1 mg/$10^8$ cells) | Tyrosine (10 mg/$10^8$ cells) |
|---|---|---|---|---|
| $10^7$ | N.D. | 4/10 | 5/10 | 9/10 |
| $10^6$ | 8/8* | 0/8 | 0/8 | 3/8 |
| $10^5$ | 8/8 | 0/8 | 0/8 | 0/8 |
| $10^4$ | 6/8 | 0/8 | 0/8 | 0/8 |

* Number of tumours observed/number of mice/group.

* Dinitrophenylation by exposure to 0·025 μg/ml DNFB.

of mice as described earlier. Although immunization with x-ray-killed tumour cells resulted in diminished tumour formation, immunization with dioxane-treated or tyrosylated tumour cells did not prevent tumour formation (Table X).

### DISCUSSION

Our attempts to enhance rejection of tumour cells by pre-immunization with hapten-coated cells were unsuccessful. This was not the result of decreased cell viability, since this factor was not affected by DNP attachment, nor does it seem to be due to the loss of antigens from the cell surface, as can be judged from the antiglobulin test performed with tyrosylated cells (Table VIII). Loss of some antigenic determinants, not necessarily H-2 antigens, however, cannot be excluded, since the ability of TYR cells to produce tumours in mice was drastically diminished. It may be that different methods of immunization are necessary for the enhancement of immunogenicity.

Studies on the ability of the coupling of DNP to mouse tumour cells to enhance immunogenicity in syngeneic recipients have been reported recently by Martin *et al.* (1971), whose studies differed from ours in that the DNP was coupled to cell surfaces in the form of 2-4 dinitrophenylaminocaproate. In addition, they made no attempt to investigate the *viability* of hapten-coupled cells or to assess the effect of hapten concentration on cell membranes on the immunogenicity of tumour cells. The latter point is of particular importance, since Wolf has shown that immunogenic tumour cells are no longer capable of protecting mice from subsequent challenge with viable tumour cells if the immunizing cells have been heavily coated with DNP (Wolf *et al.*, 1970). Nevertheless, Martin *et al.* (1971) were able to detect slightly but significantly increased cell mediated cytotoxicity for native tumour cells in the spleens of animals immunized with DNP-coated cells. They did not investigate whether such *in vitro* evidence of immunity in DNP-tumour cell immunized animals correlated with an increased ability of such mice to reject tumours *in vivo*. Thus, although our results are at variance with theirs, the difference may simply reflect the discrepancy between the methods employed. Ultimately, however, the significance of any similar attempts to enhance the rejection of tumour cells by pre-immunization with hapten-coated cells will be best established by evidence of an *in vivo* expression of the immune state.

The fact that exposure of both tumour cells and normal spleen cells to DNFB yielded cells that were comparable both in the number of DNP molecules coupled, and in the subsequent viability of the DNP-coated cells, suggests that the effects of DNFB on tumour cells is not unique to special membrane adaptations related to their oncogenic properties. The methods of hapten attachment which we have described may be of value to other workers interested in pursuing this problem.

The authors are grateful to Professor N. A. Mitchison for valuable advice and criticism.

Beverley, P. C. L. & Simpson, E. (1970) Humoral Responses to Tumour Xenografts in ALS-treated Mice. *Int. J. Cancer*, **6**, 415.

Brownstone, A., Mitchison, N. A. & Pitt-Rivers, R. (1966) Chemical and Serological Studies with an Iodine-containing Synthetic Immunological Determinant 4-Hydroxy-3-iodo-5-nitrophenylacetic Acid (NIP) and Related Compounds. *Immunology*, **10**, 465.

Martin, W. J., Wunderlich, J. R., Fletcher, F. & Inman, J. K. (1971) Enhanced Immunogenicity of Chemically-coated Syngeneic Tumor Cells. *Proc. natn. Acad. Sci. U.S.A.*, **68**, 469.

Mitchison, N. A. (1970) Immunologic Approach to Cancer. *Transplantn. Proc.*, **2**, 92.

Rimon, A. & Sela, M. (1966) Chemical Modification of Human Erythrocytes by Attachment of Tyrosine and Alanine Peptides. *Biochim. biophys. Acta*, **124**, 408.

Sela, M. & Arnon, R. (1960) Studies on the Chemical Basis of the Antigenicity of Proteins, 1. Antigenicity of Polypeptidyl Gelatins. *Biochem. J.*, **75**, 91.

Wolf, A., Parry, D. M. & Barfoot, R. K. (1970) Loss of Weak Antigenicity of Lymphoma Cells Following Treatment with Difluorodinitrobenzene. *Transplantation*, **10**, 340.